# Pituitary ​​​​Apoplexy​​ With Pituitary Macroadenoma in a Patient With ​​Asymptomatic ​​​COVID-19: ​​A Case ​​​Report

**DOI:** 10.7759/cureus.32810

**Published:** 2022-12-22

**Authors:** Hadi S Alyami, Muhannad M Al Wadany, Abdulelah S Almousa, Ethar A Khawaji, Abdulrahman M Almousa, Mohammed A Albaqshi, Ahlam S Alharbi

**Affiliations:** 1 General Practice, King Faisal University, Hofuf, SAU; 2 General Practice, Jazan University, Jazan, SAU; 3 General Practice, University of Wrocław, Wroclaw, POL; 4 Family Medicine, Primary Health Care Centers, Riyadh, SAU

**Keywords:** pituitary apoplexy, covid-19, subarachnoid hemorrhage, headache, computed tomography, magnetic resonance imaging, case report

## Abstract

Pituitary apoplexy is a rare and potentially life-threatening condition that usually occurs in the setting of a pre-existing pituitary tumor, which may be undiagnosed. There are a growing number of reports describing the pituitary apoplexy associated with coronavirus disease 2019 (COVID-19). We present the case of a 41-year-old man who presented with a gradually worsening headache for four days. It was a bilateral frontal headache of sharp quality with no radiation. He scored the headache as 9 out of 10 on the 10-point severity scale. He had no previous episodes of similar headaches. Fundoscopic examination revealed bilateral optic disc blurring suggestive of papilledema and cranial nerves examination revealed bilateral hemianopia. The patient was admitted for further investigation and management. As part of the admission protocol, the patent underwent a nasopharyngeal swab for severe acute respiratory syndrome coronavirus 2 (SARS-CoV-2), which yielded positive results. Computed tomography demonstrated a large solid intrasellar mass with areas of high density suggesting hemorrhage along with a small amount of subarachnoid hemorrhage space in the left parietal lobe. The findings were consistent with pituitary apoplexy in the setting of pituitary macroadenoma. Intravenous hydrocortisone was administered. The patient underwent transsphenoidal surgical resection of the pituitary tumor, which resulted in significant improvement in the patient's symptoms. Pituitary apoplexy is a rare condition. The case suggests that COVID-19 may predispose to the development of pituitary apoplexy.

## Introduction

Pituitary apoplexy is a rare and potentially life-threatening condition in which there is a hemorrhage or infarction of the pituitary gland. This usually occurs in the setting of a pre-existing pituitary tumor, which may be undiagnosed. Certain conditions predispose to the occurrence of pituitary apoplexy [1]. These conditions include pregnancy (Sheehan syndrome), coagulopathy, and some medications such as cabergoline, bromocriptine, and vardenafil. Neurological complications of coronavirus disease 2019 (COVID-19) are common. There are a growing number of reports describing the pituitary apoplexy associated with COVID-19 [2-4]. However, the connection between pituitary apoplexy and COVID-19 is quite debatable due to the presence of confounding factors such as anticoagulant therapy [3-4]. Here, we present the case of pituitary apoplexy with undiagnosed pituitary macroadenoma in a patient with asymptomatic COVID-19.

## Case presentation

A 41-year-old man was referred to our emergency department with a gradually worsening headache for four days. The headache was in the frontal region bilaterally and sharp in nature. It was associated with mild nausea. The patient was unable to identify any exacerbating factors for headaches. The headache was partially relieved with simple analgesics such as paracetamol. He scored the headache as 9 out of 10 on the 10-point severity scale. He had no previous episodes of similar headaches. There was no history of head trauma. There was no history of an altered level of consciousness associated with the headache. The past medical history of the patient was remarkable for celiac disease. His surgical history included elective laparoscopic cholecystectomy for recurrent episodes of biliary colic. The patient had no known drug or food allergy. He was a heavy smoker but had quit smoking the last four years. He never consumed alcohol or used recreational drugs. The patient was not on any regular medications. His family history was significant for ulcerative colitis in all his siblings.

On examination, the patient was restless. Vital signs revealed tachycardia with a pulse rate of 110 beats per minute. Other vital signs, including temperature, blood pressure, respiratory rate, and oxygen saturation, were within the normal ranges. Neurological examination of the upper and lower limbs revealed normal tone and power. The gait examination was normal. However, fundoscopic examination revealed bilateral optic disc blurring suggestive of papilledema. Additionally, the examination of the cranial nerves revealed bilateral hemianopia. The patient was admitted for further investigation and management. As part of the admission protocol, the patient underwent a nasopharyngeal swab for SARS-CoV-2, which yielded positive results. However, the chest radiograph was normal. Initial hematological and basic biochemical laboratory investigations revealed normal results

**Figure 1:**
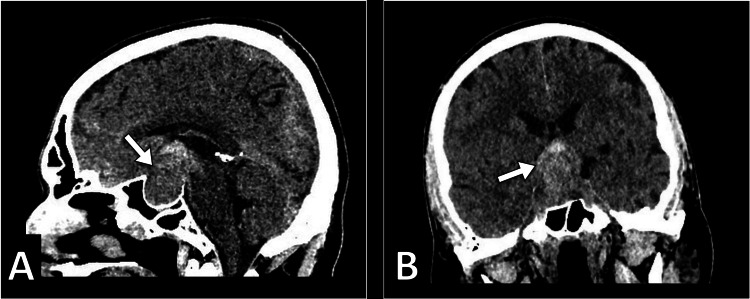
Sagittal (A) and coronal (B) CT images show a large intrasellar mass (arrow) with areas of high density suggestive of hemorrhage in the pituitary gland CT: computed tomography

**Figure 2:**
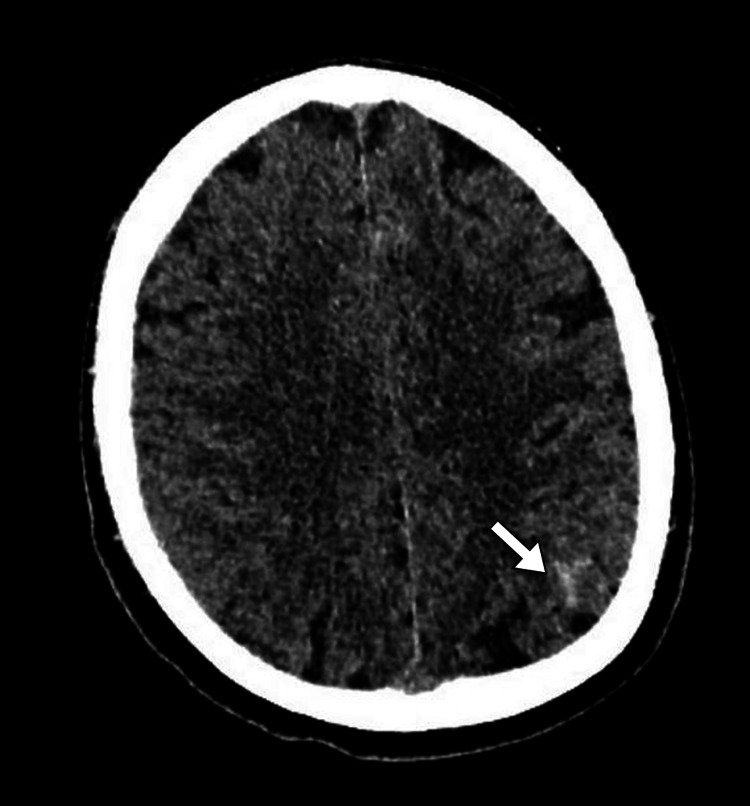
Axial head CT image shows subarachnoid hemorrhage (arrow) in the left parietal lobe CT: computed tomography

**Figure 3:**
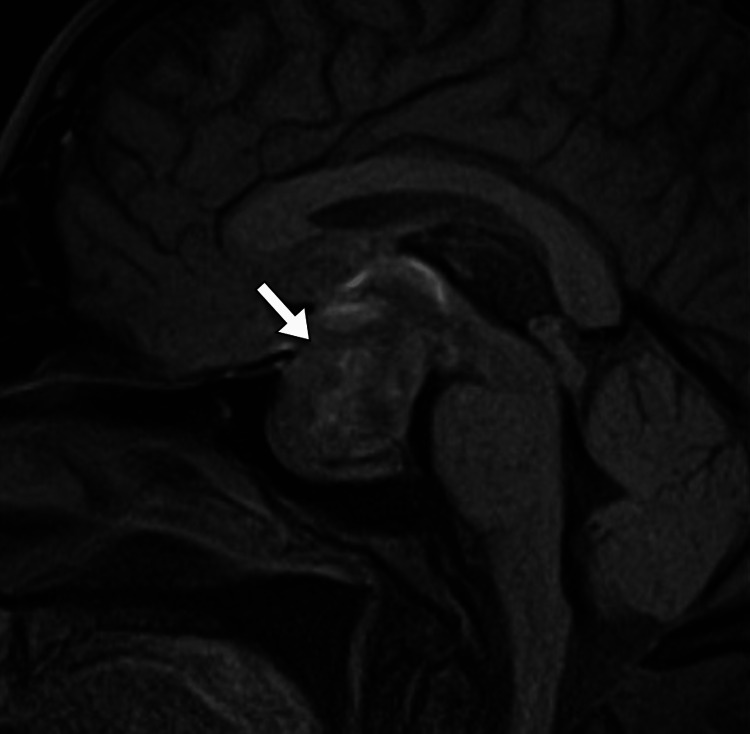
Sagittal MRI T1-weighted image shows a large intrasellar mass (arrow) enlarging the sella turcica and expanding into the suprasellar cistern MRI: magnetic resonance imaging

**Figure 4:**
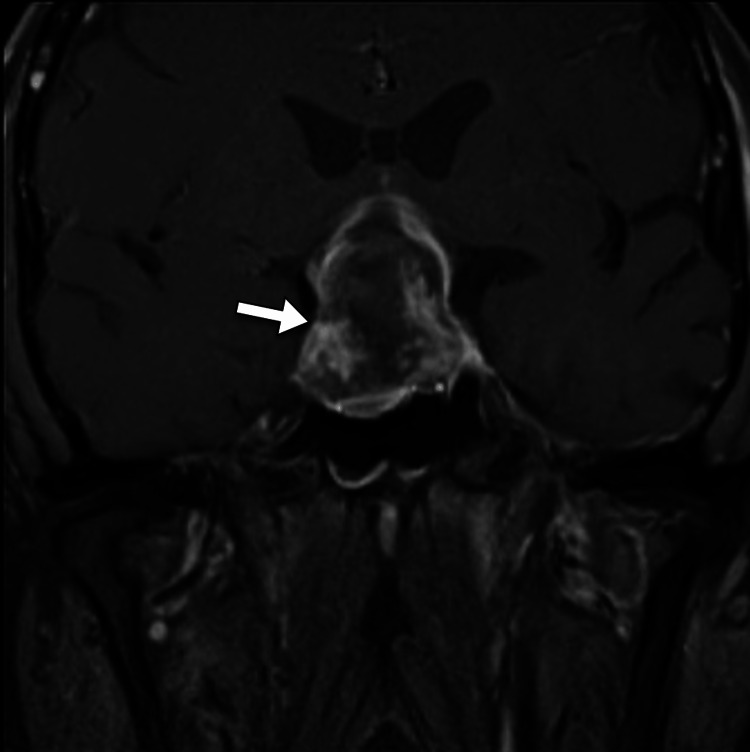
Coronal MRI post-contrast T1-weighted image shows a heterogeneously enhancing intrasellar mass (arrow) MRI: magnetic resonance imaging

The patient underwent head computed tomography to exclude any space-occupying lesion. The scan demonstrated a large solid intrasellar mass with areas of high density suggesting hemorrhage. The mass was associated with the enlargement of the sella turcica and extends superiorly into the suprasellar cistern (Figure 1). A small amount of hemorrhage was noted in the subarachnoid space in the left parietal lobe (Figure 2). Subsequently, the patient underwent magnetic resonance imaging for further evaluation of the intrasellar lesion. The scan showed a large pituitary mass with heterogeneous signal intensity. It has areas of intrinsic high intensity on T1-weighted images, suggestive of blood products. The mass was associated with the remodeling of the sella turcica, had a suprasellar extension, and caused compression of the optic chiasm. Normal flow voids are noted within the internal carotid arteries (Figures 3-4).

The aforementioned imaging features were consistent with pituitary apoplexy, undiagnosed pituitary macroadenoma, and small subarachnoid hemorrhage. The patient received a 100 mg bolus of hydrocortisone bolus with an additional 50 mg every six hours. The vital signs post-treatment included a pulse rate of 90 beats/minute, blood pressure of 130/88 mmHg, respiratory rate of 14 breaths/min, and normal temperature of 37.1 degrees. The diagnosis was discussed with the patient and the decision to proceed with decompressive surgery was made. The patient underwent transsphenoidal surgical resection of the pituitary tumor. The histopathological examination revealed monomorphic eosinophilic cells on hematoxylin and eosin stain and showed a strong and diffuse reaction of growth hormone expression of growth hormone on immunohistochemistry. These findings were consistent with a pituitary somatotroph adenoma. No complications occurred during the surgery. Subsequently, the patient had a remarkable improvement in his symptoms within one week of the surgery with regular follow-ups at the clinics. He was discharged after 10 days of hospitalization and was advised to have a monthly follow-up visit for one year. His hormonal replacement therapy included hydrocortisone and thyroxine. At the one-month follow-up visit, the patient reported no active complaints.

## Discussion

We reported a case of pituitary apoplexy in a man with undiagnosed pituitary macroadenoma who had positive COVID-19 test results. While the occurrence of pituitary apoplexy in the setting of COVID-19 can be a coincidence, there has been a growing number of case reports suggesting a potential link between these conditions [4].

Pituitary apoplexy is a rare condition. In more than 50% of pituitary apoplexy cases, no precipitating factors could be identified [1]. The known precipitating factors include angiography, cardiac surgery, head trauma, anticoagulation, and dynamic hormonal tests [1]. The reported cases of pituitary apoplexy with COVID-19 suggested that severe infections could lead to a marked inflammatory response that may result in vascular dysfunction predisposing to the development of pituitary apoplexy [2-4]. However, not all the described cases involved severe COVID-19 disease, indicating that vascular dysfunction might be an insufficient explanation for pituitary apoplexy in COVID-19. Alternatively, since the SARS-CoV-2 virus enters cells via the angiotensin-converting enzyme-2 (ACE-2) receptors, which are abundant in pituitary cells, the possibility of direct cell damage could be a more plausible pathophysiological explanation for pituitary apoplexy in COVID-19 [4]. It is interesting to note that some cases of pituitary apoplexy in COVID-19 did not have a pre-existing pituitary macroadenoma [5].

Pituitary apoplexy can be difficult to diagnose clinically. A computed tomography scan is an essential examination for patients with severe headaches, as in the present case. It aims to exclude other acute cerebrovascular events, specifically subarachnoid hemorrhage, and can demonstrate the intrasellar mass [1]. The diagnosis can be accurately made by magnetic resonance imaging. The optimal management of pituitary apoplexy is controversial [2,3]. Some authors advocate a conservative approach for selected patients while others adopt surgical decompression for all patients with pituitary apoplexy [1]. In the present case, surgical decompression was used because the patient had visual impairment.

## Conclusions

Pituitary apoplexy is a rare condition. The case suggests that COVID-19 may predispose to the development of pituitary apoplexy. Hence, physicians should be aware of this condition and consider neuroimaging in the appropriate settings to allow for its prompt diagnosis. Pituitary apoplexy can be a life-threatening condition due to corticotropic deficiency. Immediate administration of glucocorticoid is crucial once the diagnosis is confirmed.

## References

[1] Briet C, Salenave S, Bonneville JF, Laws ER, Chanson P (2015). Pituitary apoplexy. Endocr Rev.

[2] Liew SY, Seese R, Shames A, Majumdar K (2021). Apoplexy in a previously undiagnosed pituitary macroadenoma in the setting of recent COVID-19 infection. BMJ Case Rep.

[3] Ghosh R, Roy D, Roy D, Mandal A, Dutta A, Naga D, Benito-León J (2021). A rare case of SARS-CoV-2 infection associated with pituitary apoplexy without comorbidities. J Endocr Soc.

[4] Solorio-Pineda S, Almendárez-Sánchez CA, Tafur-Grandett AA, Ramos-Martínez GA, Huato-Reyes R, Ruiz-Flores MI, Sosa-Najera A (2020). Pituitary macroadenoma apoplexy in a severe acute respiratory syndrome-coronavirus-2-positive testing: causal or casual?. Surg Neurol Int.

[5] Bordes SJ, Phang-Lyn S, Najera E, Borghei-Razavi H, Adada B (2021). Pituitary apoplexy attributed to COVID-19 infection in the absence of an underlying macroadenoma or other identifiable cause. Cureus.

